# Rare Case of Ileocecal Obstruction Secondary to Endometriosis Presenting for the First Time

**DOI:** 10.7759/cureus.17074

**Published:** 2021-08-10

**Authors:** Sushruth Shetty, Deepak Varma

**Affiliations:** 1 Surgical Gastroenterology, Mazumdar Shaw Cancer Centre, Bengaluru, IND; 2 Gastrointestinal Surgery, Health City Cayman Islands, Grand Cayman, CYM

**Keywords:** intestinal endometriosis, diagnostic laparoscopy, abdomen pain, right hemicolectomy, ileocecal obstruction

## Abstract

Though endometriosis involving the intestines is well known, it causing ileocecal obstruction is a rare presentation. Etiology for ileocecal obstruction may not be known in all the cases preoperatively and may sometimes need resection and histopathology for diagnosis. Here we present a case of endometriosis presenting for the first time as an ileocecal obstruction in a 39-year-old lady who presented to us with complaints of intermittent abdominal pain. Contrast CT scan of the abdomen showed terminal ileal stricture and wall thickening. She underwent diagnostic laparoscopy, which showed dilated distal small bowel loops with suspicious stricturing growth at the terminal ileum and ileocecal valve region. A formal laparoscopic right hemicolectomy was done and post-operative histopathology revealed endometriosis with fibrosis, causing a luminal obstruction. In conclusion, endometriosis should be considered as a rare differential in patients presenting with ileocecal obstruction and having inconclusive features on imaging, endoscopic or biopsy, especially in women of childbearing age.

## Introduction

Intestinal obstruction at the ileocecal region is most commonly caused by aetiologies like tuberculosis, Crohn's disease, malignancy including lymphomas, radiation enteritis, or adhesions. Though endometriosis involving the intestines is known, these causing ileocecal obstruction is a rare presentation and is found only as case reports [1-4]. Endometriosis is the presence of endometrial-type mucosa outside the uterine cavity. Menstrual irregularities, chronic pelvic pain, and urinary disturbances are the most frequently reported symptoms. Standard diagnosis is carried out by direct visualization and histological examination of lesions [5]. The most common site of extragenital endometriosis is the intestinal tract, and the reported incidence ranges from 3% to 37% in patients diagnosed with endometriosis [6,7]. Evaluation of a patient with ileocecal obstruction is mainly by imaging and endoscopy with biopsy. But in many instances in patients with symptomatic obstruction, the final diagnosis is obtained only after surgical resection and histopathological examination. Here we present a rare case of a patient with ileocecal obstruction who underwent laparoscopic right hemicolectomy and post-operative histopathology revealed endometriosis.

## Case presentation

**Figure 1:**
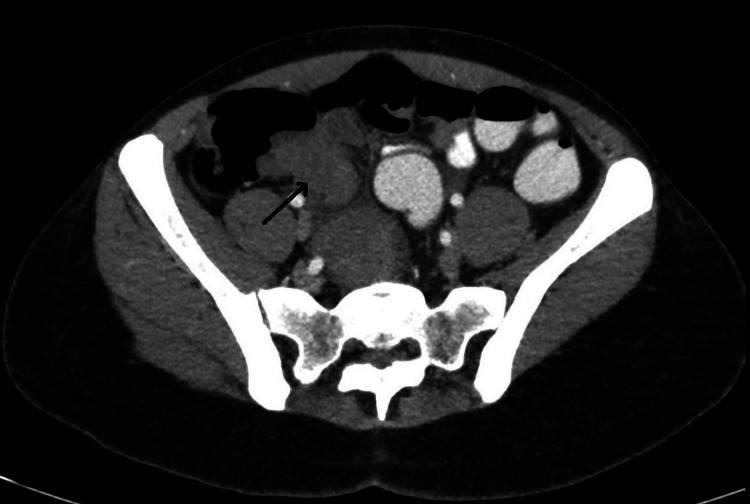
Thickening at the ileocecal valve and terminal ileum. Arrow showing terminal ileum.

**Figure 2:**
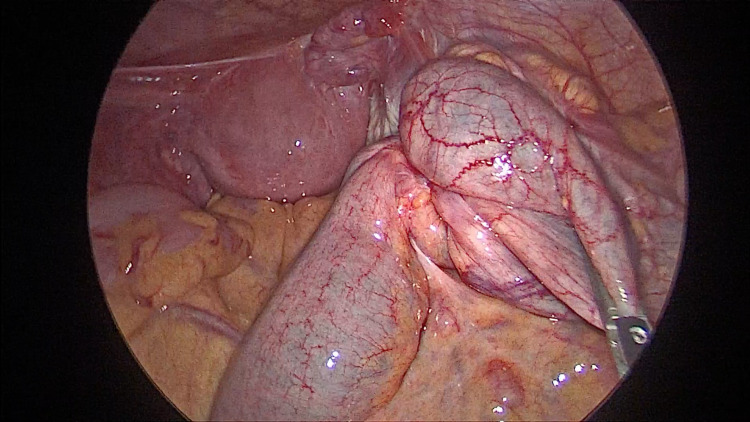
Intraoperative picture of terminal ileal obstruction and dilated proximal bowel loop. Appendix was not visualized separately.

A 39-year-old lady presented to the ED with complaints of periumbilical abdominal pain and bilious vomiting. She had been experiencing similar symptoms intermittently for the past 6-8 months. Pain aggravated after food intake. She had regular menstrual cycles and has one child born of normal term delivery. She was evaluated elsewhere for the same and all her investigations were reported to be normal. She had been managed conservatively for acid peptic disease. On examination she was in severe distress due to pain, hemodynamically stable, the abdomen was mildly distended but soft with some tenderness in the right iliac fossa and right lumbar area. A contrast CT scan of the abdomen was done which showed terminal ileal stricture with wall thickening and significant proximal dilatation of ileum with positive small bowel feces sign (Figure 1). She was further evaluated by colonoscopy, which revealed that the ileocecal valve was edematous with hypertrophied Peyer's patches and with partial intussusception of terminal ileum into the caecum. There was a significant narrowing of the distal-most ileum. Biopsies taken from the nodular areas of the mucosa showed only some nonspecific lymphoid tissue. All other tests done including tumor markers were negative. As even after 48 hours, she continued to have pain and abdominal distention, a repeat CT scan was done which showed persistent dilated small bowel loops with terminal ileal obstruction. She then underwent diagnostic laparoscopy which showed dilated distal small bowel loops with suspicious stricturing growth at the terminal ileum and ileocecal valve region (Figure 2). In view of strong suspicion of malignancy and nonavailability of frozen section examination, a formal laparoscopic right hemicolectomy with stapled, side to side, ileo-transverse anastomosis was done. Postoperatively, she had an uneventful recovery and was discharged home on postoperative day 4. On histopathology, gross examination showed cecal induration with intussusception of the distal terminal ileum into the cecum with associated obstruction of the ileocecal valve. Serial sections at the level of the cecum and distal terminal ileum showed marked fibrosis along the adjoining wall. The area of fibrosis extended partially along with the ileocecal valve and partially along the cecal wall. On microscopy and immunohistochemistry, there were features suggestive of endometriosis with PAX8 and estrogen receptors highlighting glandular epithelium nuclei and CD10 highlighting the surrounding stromal tissue.

## Discussion

The differential diagnosis for ileocecal obstruction may be varied but endometriosis is usually considered a common etiology, especially in patients without prior symptoms of endometriosis. The theory of retrograde menstruation is the most commonly accepted hypothesis for endometriosis and rectosigmoid is the most frequently involved segment of intestinal endometriosis [8,9]. The majority of the symptoms of endometriosis are cyclical, but this may not be true in all cases. Endometriosis mimicking Crohn's disease, irritable bowel syndrome, and even malignancies is known [5,6]. Deep infiltrating types of intestinal lesions can cause complications and these usually warrant surgical resection [8]. Ileocecal involvement causing intestinal obstruction is very rare and pre-operative diagnosis may not be possible in all patients. The final diagnosis is based on histopathology and the presence of endometrial epithelial and stromal cells at ectopic sites. Studies also have proved the pro-fibrotic nature of these lesions which may be of the important mechanism by which it may cause intestinal obstruction [10]. This results in strictures which on imaging or diagnostic laparoscopy may be difficult to differentiate from neoplastic lesions [2,7]. Medical management usually fails in these patients and hence almost always warrants resection [9]. Also in these patients, it is important to do a complete diagnostic laparoscopy to rule out other focuses of endometriosis, especially those involving the intestines, as they are known to be multicentric and may cause complications if left behind [2,11].

## Conclusions

Intestinal obstruction secondary to endometriosis is rare and that too as the first presentation of endometriosis is very rare. It should be considered as a differential in patients presenting with ileocecal obstruction having inconclusive imaging, endoscopic and biopsy features, especially in women of childbearing age group.
